# Class I PI3K inhibitor ZSTK474 mediates a shift in microglial/macrophage phenotype and inhibits inflammatory response in mice with cerebral ischemia/reperfusion injury

**DOI:** 10.1186/s12974-016-0660-1

**Published:** 2016-08-22

**Authors:** Po Wang, Yating He, Daojing Li, Ranran Han, Guiyou Liu, Dexin Kong, Junwei Hao

**Affiliations:** 1 Department of Neurology, Tianjin Medical University General Hospital, Tianjin, 300052 China; 2 Department of Neurology, Baotou Central Hospital, Baotou, Inner Mongolia 014000 China; 3 Genome Analysis Laboratory, Tianjin Institute of Industrial Biotechnology, Chinese Academy of Sciences, Tianjin, 300000 China; 4 Tianjin Key Laboratory on Technologies Enabling Development of Clinical Therapeutics and Diagnostics, School of Pharmacy, Tianjin Medical University, Tianjin, 300070 China; 5 Tianjin Neurological Institute, Tianjin Medical University General Hospital, Tianjin, 300052 China

**Keywords:** Cerebral ischemia reperfusion injury, Inflammation, ZSTK474, Microglia/macrophages, PI3K/AKT/mTORC1 pathway

## Abstract

**Background:**

Microglia/macrophages play a critical role in the inflammatory and immune processes of cerebral ischemia/reperfusion injury. Since microglia/macrophages can reversibly shift their phenotype toward either a “detrimental” or a “restorative” state in the injured central nervous system (CNS), compounds mediate that shift which could inhibit inflammation and restore the ability to alleviate cerebral ischemia/reperfusion injury would have therapeutic potential.

**Methods:**

Transient middle cerebral artery occlusion was induced in male C57BL/6 mice. Mice were randomly separated into a sham-operated group, a control group, and a ZSTK474-treated group. We investigated the effect of ZSTK474 by assessing neurological deficits, infarct volume, and histopathological changes. We then determined the potential mechanism by immunofluorescent staining, quantitative real-time polymerase chain reaction (PCR), and Western blot analysis. The Tukey’s test or Mann–Whitney *U* test was used to compare differences among the groups.

**Results:**

ZSTK474 alleviated neurological deficits and reduced infarct volume in the cerebral ischemia/reperfusion injury model. Presumably, ZSTK474 shifted the phenotype of microglia/macrophages to a restorative state, since this treatment decreased the secretion of pro-inflammatory factors and advanced the secretion of anti-inflammatory factors. These neuroprotective properties of ZSTK474 may be mediated by the phosphoinositide 3-kinase (PI3K)/AKT/mammalian target of rapamycin complex 1 (mTORC1) pathway.

**Conclusions:**

ZSTK474 can mediate a shift in microglia/macrophage phenotype and inhibit the inflammatory response in cerebral ischemia reperfusion injury of mice. These effects appeared to ensue via the PI3K/AKT/mTORC1 pathway. Therefore, ZSTK474 may represent a therapeutic intervention with potential for circumventing the catastrophic aftermath of ischemic stroke.

**Electronic supplementary material:**

The online version of this article (doi:10.1186/s12974-016-0660-1) contains supplementary material, which is available to authorized users.

## Background

Stroke is a devastating illness, second only to cardiac ischemia as a cause of human death worldwide [1]. Eighty-seven percent of strokes are ischemic [1]. However, no treatment has been effective thus far in preventing brain damage in such cases. The only agent now available for these patients is the enzyme, tissue plasminogen activator (TPA), but its effectiveness is limited to a short time interval after the ischemic event, when the reperfusion of brain tissue can be quickly restored [2].

When blood flow is interrupted, the brain becomes ischemic which leads to a series of disastrous effects such as lack of nutrient delivery and accumulation of toxic substances. The brain is exceedingly vulnerable to neurologic damage because it does not store oxygen and glycogen but instead relies on delivery of those substances by the blood. Re-establishment of blood flow to ischemic areas is now known as the most effective strategy for treating ischemic stroke [3]. But the process of this reperfusion can increase damage to ischemic brain tissue. Therefore, the development of a therapy that targets on reperfusion injury in ischemic stroke is essential, as is a means of prolonging the treatment time frame beyond the period of TPA’s effectiveness.

Inflammation plays a key role in the pathogenesis of stroke. This process partly depends on pro-inflammatory and anti-inflammatory factors released by immune cells, such as microglia and macrophages. When reperfusion is used as a treatment, the ensuing restoration of blood flow induces a dramatic response from activated microglia/macrophages as well as severely injurious activity mediated by pro-inflammatory cytokines, such as tumor necrosis factor (TNF-α) and interleukins (e.g., IL-6, IL-1β). The result is exacerbation of tissue damage. One novel strategy in treating acute cerebral ischemia and reperfusion injury is to dampen this inflammation indirectly by adjusting microglial/macrophage phenotype, which in turn “short circuits” a cascade of inflammatory responses.

In the normal healthy brain, microglia acts as surveillance “sensors.” They endlessly monitor and respond to changes in the brain’s microenvironment. Microglia and macrophages, which can reversibly shift their functional phenotype through a multitude of patterns in response to changes of the microenvironment [4], have the capacity to elicit a nonspecific innate immune response when ischemic reperfusion injury occurs [5]. However, this immune response induces restorative as well as detrimental effects. In the detrimental form, they release pro-inflammatory mediators that can hinder CNS repair, leading to more serious tissue damage. The restorative form of microglia/macrophages promotes brain regeneration and recovery by clearing cell debris, resolving local inflammation, and releasing protective factors. These characteristics of microglia/macrophages mandate their likely value as a novel target for treating cerebral ischemia. Therefore, a major thrust of research on ischemic stroke is to find methods to suppress the deleterious effects of microglia/macrophages without compromising neurovascular repair and remodeling [6].

ZSTK474 [2-(2-difluoromethylbenzimidazol-1-yl)-4, 6-dimorpholino-1, 3, 5-triazine] is one of PI3K inhibitors that have promising antitumor effects while lacking observable toxicity. Since this compound has been tested as an anticancer drug in phase I clinical trials [7–10], some basic knowledge has been gained about its effects in humans. Previous research has demonstrated, for example, that ZSTK474 has anti-inflammatory properties which could protect mice from collagen-induced arthritis and multiple sclerosis, both of which are autoimmune inflammatory diseases [11–13]. Since those findings indicate that ZSTK474 has anti-inflammatory and immunoregulatory potential, it is reasonable to hypothesize that ZSTK474 may improve the prognosis of patients with cerebral ischemia/reperfusion injury by inhibiting the inflammatory response. In the present study, we used mice suffered from middle cerebral artery occlusion (MCAO) to mimic cerebral ischemia/reperfusion injury in humans.

## Methods

### Animals

Male C57BL/6 mice (aged 10–12 weeks) were purchased from Charles River Laboratories (Beijing, China). The mice were maintained in a temperature-controlled environment under a 12-h light and 12-h dark cycle. Normal amounts of food and water were provided. Experiments were performed according to national regulations and were approved by the Animal Experiments Ethical Committee of Tianjin Medical University General Hospital. Mice were allowed to acclimate to the conditions for 1 week.

### Induction of MCAO

Cerebral infarction was simulated by occlusion of the left middle cerebral artery using the methods reported by Longa et al. [14]. First, mice were anesthetized with the intraperitoneal (i.p.) injection of chloral hydrate (30 mg/kg). Second, a midline neck incision was made to expose the left common carotid artery, the external carotid artery, and the internal carotid artery; each of them was then isolated and ligated. A monofilament with a silicone-beaded tip (xinong, Beijing, China) was introduced into the internal carotid artery through the common carotid artery. Mild resistance to the tip’s insertion indicated the blocking of all blood flow from the posterior cerebral artery, the internal carotid artery, and the anterior artery. One hour after the occlusion of blood, the monofilament was removed and blood flow was restored. Regional cerebral blood flow (rCBF) was monitored by Laser Doppler Flowmeter (PF5010) with a flexible fiber optic probe fixed to the skull above the territory of the left middle cerebral artery (rCBF decreased by 85 %). In the sham-operated group, mice underwent the same surgical protocol except insertion of the monofilament into artery. Body temperature was maintained within a normal range (37 to 38 °C) by a temperature-controlled heating pad. Food and water were provided ad arbitrium. For analgesia, buprenorphine was administered i.p. at the dose of 0.03 mg/kg every 12 h for 24 h.

### Treatment with ZSTK474

ZSTK474 was provided by the Zenyaku Kogyo Co. (Tokyo, Japan). ZSTK474 was suspended in 5 % hydroxypropylcellulose in water as a solid dispersion. First, mice were randomly assigned to receive different doses of ZSTK474 (50, 100, 200, and 300 mg/kg) to determine the optimum dose; in our experiment, the optimum dose was 200 mg/kg (Additional file 1: Figure S1). Then mice were randomly assigned to one of three groups: a sham-operated group (phosphate-buffered saline, PBS); a control group (MCAO + PBS); a ZSTK474-treated group (MCAO + ZSTK474). In the ZSTK474-treated group, the mice were given the optimum dose of 200 mg/kg ZSTK474. In the sham-operated group and control group, mice were given an equivalent volume of PBS. All mice received that same dose daily via oral gavage beginning at 6 h after the onset of focal ischemia and continuing for two more days, i.e., for a total of 3 days.

### Assessment of neurological outcome following MCAO

Ten mice from each group were used for neurobehavioral testing. Mice underwent neurobehavioral testing at 24, 48, and 72 h after MCAO. These tests, including the modified neurological severity score (mNSS), the corner test, adhesive removal test, and foot fault test were administered as described in the following text. The observer/scorer was blinded to the experimental conditions. The mNSS [15] is a composite test of motor, sensory, reflex, and balance. Neurological function was graded on a scale of 0 to 18, with a score of 0 being normal and a score of 18 denoting maximum deficits. For the severity scores of injury, one point was recorded for the inability to perform a test or for the lack of performing a tested reflex. Therefore, higher scores reflected greater injury.

In the corner test [16], a mouse was placed between two boards, each 30 × 20 × 1 cm in size. The edges of the two boards were attached at a 30° angle with a small opening along the joint placed between the two boards to encourage the mice to enter the corner. At the beginning of a trial, the mouse was placed between the two angled boards facing the corner, halfway to the corner. When the mouse was wedged into the corner, both sides of its body were stimulated simultaneously. The mouse usually reared and turned either to the right or left. Each mouse was tested for 10 trials and the side to which it turned (right or left) when stimulated was recorded. If a mouse turned without rearing (i.e., ventral turning), the trial will not be recorded. Rather, it was repeated at the end of the session. In general, a non-ischemic mouse turned either to its left or right, whereas a mouse suffered from MCAO preferentially turned toward its non-impaired ipsilateral side. The data were expressed as the percentage of right turns as a function of the total number of turns and then these were statistically analyzed.

The adhesive removal test [17–19] assessed somatosensory and motor deficits. All mice were trained for this test before undergoing MCAO. After the mouse familiarized with the testing environment, two small pieces of adhesive paper dots were attached to the distal-radial region on the wrist of each forelimb with equal pressure. The time it took for the mouse to remove each paper dot (stimulus) from its forelimbs was recorded for 3 trials per day for each forelimb. Individual trials were separated by at least 5 min. After three consecutive days of training, all the mice were able to remove the dots. Then they were subjected to MCAO. The time a mouse took to remove the two dots from its forelimbs was recorded for statistical analysis.

In the foot fault test [20], a mouse was placed on a horizontal grid floor for 1 min. A foot fault occurred when the mouse inaccurately placed its forelimb or hindlimb and fell through one of the grid openings. The number of foot faults was recorded for statistical analysis.

### Infarct volume analysis

When the observation period ended, the mice were anesthetized with chloral hydrate (30 mg/kg, i.p.) and perfused intraventricularly with PBS (pH 7.4, 4 °C). Then, the brains were quickly acquired for histological assessment of infarct volume. The brains were sectioned into coronal sections (2 mm thick) starting from the frontal tips. The sections were stained with 1 % 2, 3, 5-tripenyltetrazolium chloride (TTC; Sigma) at 37 °C for 20 min and then fixed in 4 % paraformaldehyde at 4 °C for 2 h. With TTC, viable tissues based on intact mitochondrial function were stained deep red, whereas infarcted tissues remained unstained. The infarcted areas were evaluated using Image-Pro® Plus, v. 4.0 image analysis software (Media Cybernetics). To compensate for the effect of brain edema, the infarct volume percentage was calculated as follows: infarct area × {1 − [(ipsilateral hemisphere area − contralateral hemisphere area)/contralateral hemisphere]}. The infarct volume was expressed as a percentage of the volume of the hemisphere contralateral to the infarct [21].

### Histopathological analysis

After 72 h of reperfusion, the mice were anesthetized with chloral hydrate (30 mg/kg, i.p.) and intraventricularly perfused with PBS (pH 7.4, 4 °C). The brains were removed and fixed in 4 % paraformaldehyde overnight at 4 °C. After paraffin embedding of coronal sections (6 μm), the sections were stained with hematoxylin and eosin (H&E). Pathological and histological changes were observed through a light microscope (Olympus) at a magnification of ×20 and then documented by digital photography.

### Immunofluorescent staining and analysis

Animals were first anesthetized and then intraventricularly perfused with PBS (pH 7.4, 4 °C). The fixed brains were removed and then immersed in 30 % sucrose in PBS overnight at 4 °C. Coronal sections (8 μm) were prepared using a standard cryostat, and the cut sections were stored at −20 °C until staining. For immunostaining, sections were blocked in 10 % fetal bovine serum supplemented with 0.3 % Triton X-100 for 1 h at room temperature. Sections were next incubated with a combination of primary antibodies at 4 °C overnight. Primary antibodies included: goat anti-CD206 (1:200, R&D Systems) (double staining), rat anti-CD16/32 (1:500, BD) (double staining), rabbit anti-ionized calcium binding adaptor molecule 1 (Iba-1) (1:500, Wako) (single or double staining), and rabbit anti-glial fibrillary acidic protein (GFAP) (1:1000, Abcam) (single staining). Sections were next washed three times in PBS before incubated with two secondary antibodies for 1 h at room temperature. Images were taken using a fluorescence microscope (Olympus PX51, Olympus Corporation) and analyzed with Adobe Photoshop 5.0 software. The cell numbers were determined by counting labeled cells in three randomly selected microscopic fields across three slides in the penumbra of the ipsilateral cortex. Data are expressed as the mean number of cells per visual field for Iba-1 and GFAP and as the percentage of CD16/32^+^ or CD206^+^ cells to Iba-1^+^ cells.

### Quantitative real-time PCR

Using TRIzol reagent (Invitrogen), total RNA was extracted from the infarcted hemisphere according to the manufacturer’s instructions. We quantified the concentration of RNA with ultraviolet spectrophotometry at 260/280 nm. Complementary DNA (cDNA) was transcribed using Trans-Script First-Strand cDNA Synthesis SuperMix Kit (Transgen) in accordance with the manufacturer’s instructions. PCR was performed on an Opticon 2 Real-Time PCR Detection System (Bio-Rad) with SYBR gene PCR Master Mix (Roche). Using the 2^–ΔΔCt^ method, samples were run in triplicate and normalized to GAPDH. Then, the expression levels of messenger RNAs (mRNAs) were reported as fold changes vs. control. The primer sequences used to measure gene expression are listed in Table 1.

**Table 1:** Primers for real-time polymerase chain reaction

Gene	Primer
Gapdh	Sens: GCCAAGGCTGTGGGCAAG GT
	Revs: TCTCCAGGCGGCACGCA GA
iNOS	Sens: GGTGAAGGGACTGAGCTGTT
	Revs: ACGTTCGTTCTCTTGCA
IL-1β	Sens: ACGCTTACCATGT GAGCTG
	Revs: GCCACAGGGATTTTGTCGTT
IL-6	Sens: ACGCTTCTGGGCCTGTTGTT
	Revs: CCTGCTGCTGGTGATTCTCT
TNF-α	Sens: TCGGTCCCAACAAGGAGGAG
	Revs: GGGTTGTCACTCGAGTTTTG
CD16	Sens: TTTGGACACCCAGATGTTTCAG
	Revs: GTCTTCCTTGAGCACCTGGATC
CD86	Sens: GACCGTTGTGTGTGTTCTGG
	Revs: GATGAGCAGCATCACAAGGA
CD32	Sens: AATCCTGCCGTTCCTACTGATC
	Revs: GTGTCACCGTGTCTTCCTTGAG
CD206	Sens: AAGGAAGGTTGGCATTTGT
	Revs: CTTTCAGTCCTTTGCAAGC
Arg-1	Sens: CACCTGAGCTTTGATGTCG
	Revs: TGAAAGGAGCCCTGTCTTG
YM-1	Sens: GAGGTAATGAGTGGGTTGG
	Revs: ACGGCACCTCCTAAATTGT
IL-10	Sens: AATAAGAGCAAGGCAGTGG
	Revs: TCCAGCAGACTCAATACACA
TGF-β	Sens: GCGCTTGCAGAGATTAAAA
	Revs: GTCAAAAGACAGCCACTCA

### Quantitative enzyme-linked immunosorbent assay (ELISA) for cytokine profile

Tissue samples were taken from ischemic region of the brain. Protein levels of TNF-α, IL-6, IL-1β, IL-10, and transforming growth factor-β (TGF) were quantified using an enzyme-linked immunosorbent assay kit (Mosaic ELISA system, R&D systems) according to the manufacturer’s protocol. Determinations were performed in duplicate on individual mouse brains (*n* = 6 mice per group). Readings from each sample were normalized for protein concentration.

### Western blot analysis

Ischemic brain tissue was homogenized by sonication in ristocetin-induced platelet aggregation (RIPA) buffer, containing protease and phosphatase inhibitors (Roche Diagnostics). Protein concentration was assessed using a Protein Assay Kit (Bio-Rad). Proteins were separated on a 12 % Tris-Glycine gradient gel (Bio-Rad) from 80 to 120 V, then transferred onto nitrocellulose membranes for 1.5 h at 4 °C at 80 V. Membranes were blocked in 5 % BSA for 1 h at room temperature, incubated with primary antibody overnight at 4 °C, washed three times for 10 min each at room temperature, incubated with an HRP-coupled secondary antibody for 1 h, and washed three times for 10 min each at room temperature. All incubations were performed in TBS buffer including 0.1 % Tween-20. The following primary antibodies were used: rabbit anti-pho-p70-S6k (1:1000, Cell Signaling); rabbit anti-AKT (1:1000, Cell Signaling) and anti-p-AKT (1: 1000, Cell Signaling); mouse anti-GAPDH (1: 1000, Sigma-Aldrich). Finally, images of Western blots were captured by densitometry (Bio-Rad).

### Statistical analysis

Each experiment was repeated for three times. The data were expressed as means ± SD. Statistical analysis was performed using two-way analysis of variance (ANOVA) followed by the Bonferroni post test. One-way ANOVAs were followed by Tukey’s test or Mann–Whitney *U* tests. SPSS 17.0 (SPSS Inc.) was used for the statistical analysis. *P* < 0.05 was considered statistically significant.

## Results

### ZSTK474 decreases neurological defects, prevents histopathological changes, and reduces infarct volume

**Fig. 1:**
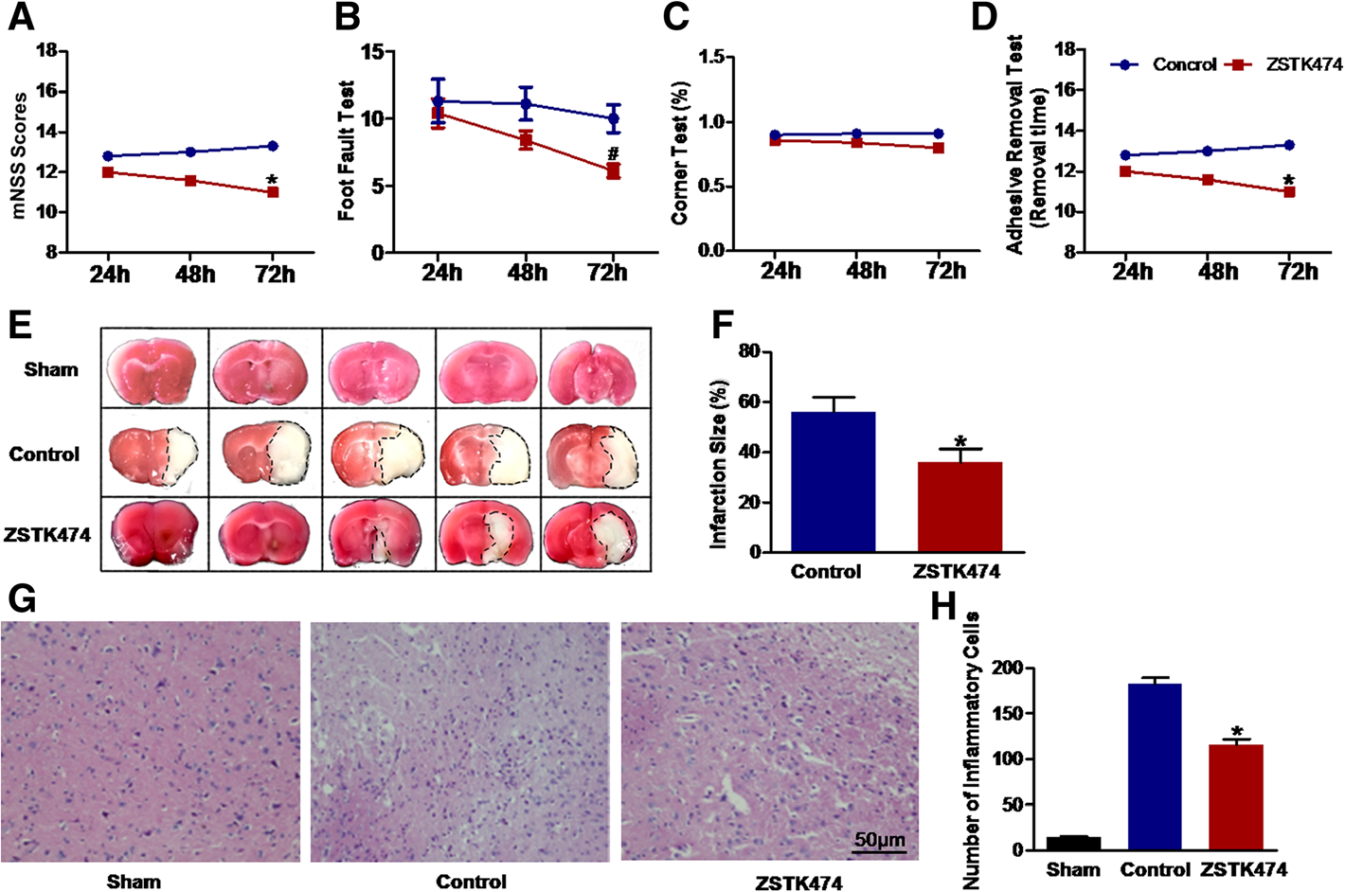
ZSTK474 alleviates neurological deficits and reduces infarct volume in a model of ischemic reperfusion. **a**–**d** Neurological deficits were evaluated at 24, 48, and 72 h after reperfusion. ZSTK474 markedly alleviate the modified neurological severity scores, adhesive removal test, and foot fault test compared to mice in the control group. There was no obvious change in corner test outcomes between ZSTK474-treated and control groups. **e** Representative TTC-stained brain sections of mice treated with 200 mg/kg ZSTK474 after MCAO (samples acquired at 72 h after stroke). **f** Quantitative analysis of the percentage of brain infarct volume. ZSTK474 treatment reduced the percentage of brain infarct volume to a level of statistically significant difference compared to the control group. **g** H&E staining showed that neurons had normal morphologic features in the sham-operated group. The MCAO group presented with multiple vacuolated interspaces, infiltration of inflammatory cells, and dead neurons. ZSTK474 significantly ameliorated the damage caused by reperfusion injury. **h** Quantitative analysis of the number of inflammatory cells in G. Data expressed as means ± SD; ^#^ *P* < 0.01, *P < 0.05, vs. control group; *n* = 6–10 per group

In mice subjected to MCAO, treatment with ZSTK474 was tested at dosages of 50, 100, 200, and 300 mg/kg. Since the 200 mg/kg dose produced significant improvement and no obvious toxic effects (*P* < 0.01) (Additional file 1: Figure S1), mice were treated with ZSTK474 at a dose of 200 mg/kg/day daily for three post-MCAO days during the remaining experiments of this study. Neurological function was examined in mice suffered from MCAO followed by 24, 48, and 72 h of reperfusion. In the ZSTK474 group, neurological function scores were significantly better than the control group except the corner test, as shown in Fig. 1a–d.

Compared with control group (MCAO + PBS), the ZSTK474-treated group (MCAO + ZSTK474) had smaller infarct volumes (*P* < 0.05) (Fig. 1f). After sectioning of brain tissues, representative histological sections of the brain showed that normal regions unaffected by the stroke were stained red, whereas the infarcted areas were pale in color (Fig. 1e). No signs of infarction were noted in mice in the sham-operated group. By using TTC staining, the infarct areas of brains were clearly visible.

Subsequently, brain tissues were stained with H&E to detect neuronal loss and vacuolated interspaces. Brain tissues were acquired from mice subjected to 1 h of ischemia followed by 72 h of reperfusion. In brain slices from the control group, no intact neurons were present in ischemic areas (Fig. 1g). By contrast, the same areas in brain slices of the ZSTK474-treated group showed only partial neuronal loss, and a few neurons remained intact in the vacuolated spaces (Fig. 1g). Quantitative analysis indicated that the total number of infiltrating cells had significantly decreased in the ZSTK474-treated group compared to controls in the ischemic penumbra (*P* < 0.05) (Fig. 1h).

### ZSTK474 inhibits the expression of Iba-1 and GFAP in the ischemic penumbra

**Fig. 2:**
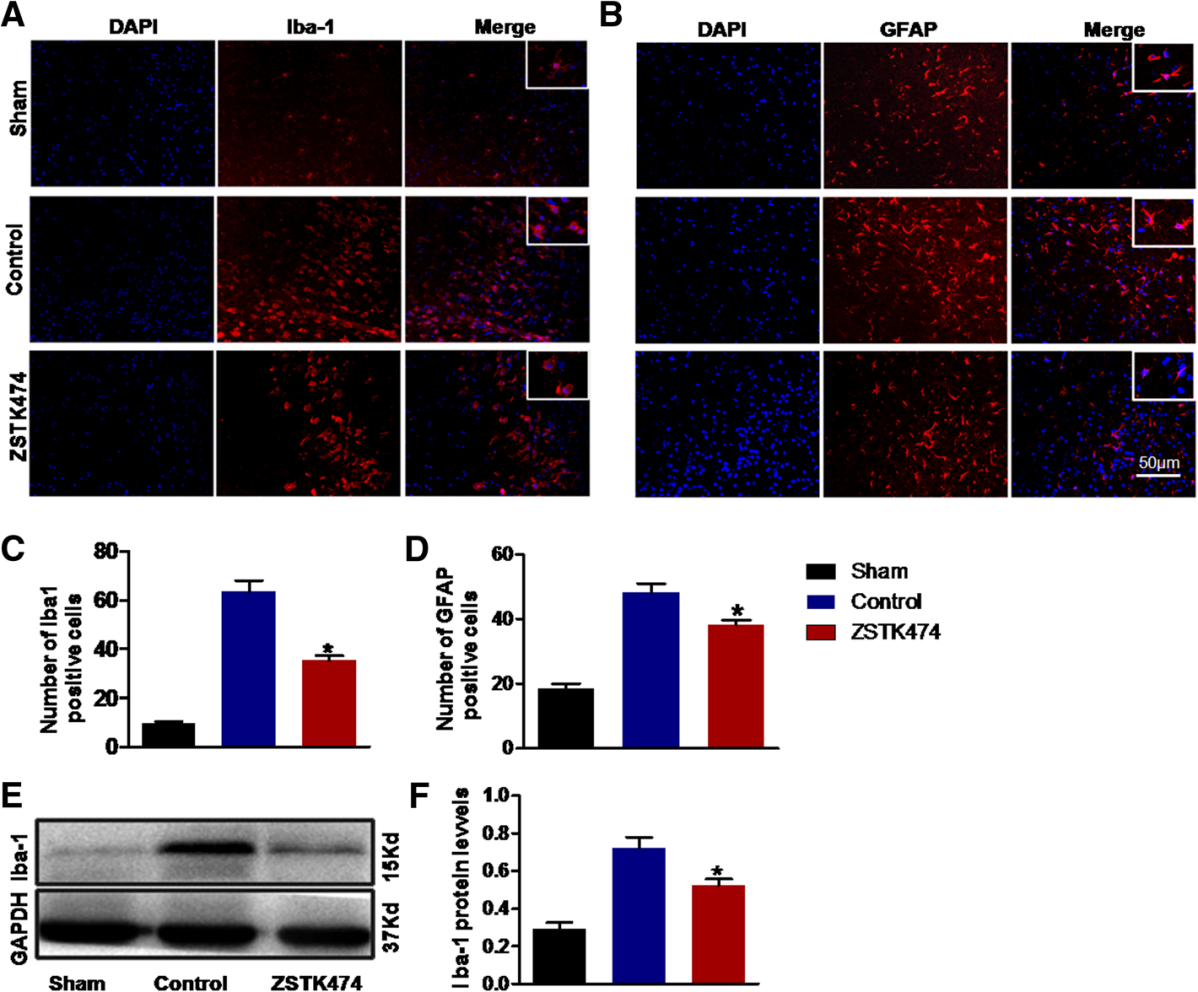
ZSTK474 inhibits the expression of Iba-1 and GFAP at 72 h after cerebral reperfusion injury. GFAP and Iba-1 are specific markers of microglia/macrophages and astrocytes. **a**, **b** The number of microglia/ macrophages and astrocytes increased as demonstrated by immunofluorescent staining. However, ZSTK474 administration decreased the number of Iba-1-stained and GFAP-stained cells. **c**, **d** Quantification of microglial/macrophage and astrocyte numbers of A, B. **e**, **f** Protein levels of Iba-1 were dramatically lower in the ZSTK474-treated group, as demonstrated by Western blotting. Magnification: ×20 in A and B. Data in graphs are expressed as means ± SD; ^#^ *P* < 0.001, *P < 0.05, vs. control group; *n* = 3

Since ischemia/reperfusion can stimulate microglia/macrophages and astrocytes [22, 23], we investigated such changes by using immunofluorescence to detect their respective markers, Iba-1 and GFAP. As shown in Fig. 2, the microglia/macrophage in the sham group was highly ramified, with their cells exhibiting long increased and branches of Iba-1-stained microglia/macrophages became short, retracted, and thick. Compared with the control group, the ZSTK474-treated group had decreased microglial/macrophage numbers and showed smaller cellular bodies (*P* < 0.001) (Fig. 2a, c). A similar pattern of results was obtained with immunostaining for GFAP, which specifically identifies astrocytes (*P* < 0.05) (Fig. 2b, d).

In Western blots, the protein level of Iba-1 increased significantly 72 h after ischemia reperfusion. In contrast, this trend was obviously suppressed in the ZSTK474-treated group (*P* < 0.05) (Fig. 2e, f). These data indicate that ZSTK474 inhibited the expression of Iba-1 in the ischemic penumbra.

### ZSTK474 shifts the phenotype of microglia/macrophages to a restorative state

**Fig. 3:**
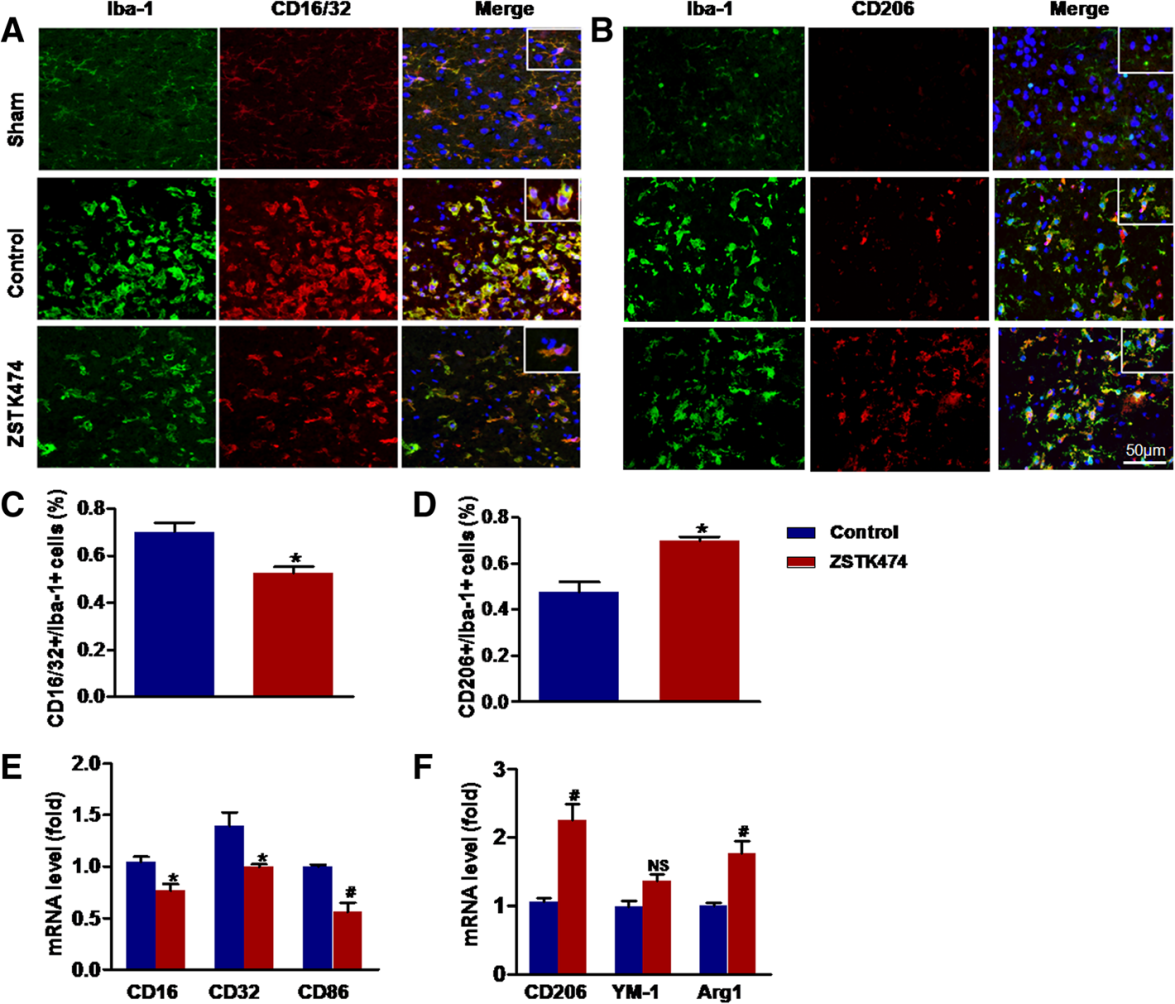
Double immunofluorescent staining for the microglial/macrophage marker Iba-1 and detrimental or restorative state markers. Brain sections were prepared 72 h after MCAO. **a** Double immunofluorescent staining for CD16/32 (*red*; detrimental state marker) and Iba-1 (*green*). **b** Double immunofluorescent staining for CD206 (*red*; restorative state marker) and Iba-1 (*green*). **c** Quantification of the percentage of CD16/32^+^/Iba-1^+^ cells. **d** Quantification of the percentage of CD206^+^/Iba-1^+^ cell. **e**, **f** PCR was performed to quantify the mRNA expression of detrimental state markers (CD16, CD32, CD86) and restorative state markers (CD206, YM-1, Arg1) in the ipsilateral cortex 72 h after MCAO. Data in graphs are expressed as means ± SD; ^#^ *P* < 0.001, *P < 0.05 vs. control group; *n* = 3–6

Microglial/macrophage phenotypes can be distinguished by their expression of characteristic surface marker genes. In double immunofluorescence staining, the expression of CD16/32, a marker of the detrimental state, was strongly present in Iba1^+^ cells (microglia/macrophages) in MCAO mice. That expression was significantly reduced in the ZSTK474-treated group (*P* < 0.05) (Fig. 3a, c). In contrast, co-expression of the restorative state markers, CD206 and Iba1, was higher in the ZSTK474-treated group compared with the control group (*P* < 0.05) (Fig. 3b, d). These results suggest not only that microglia/macrophages largely transform to a detrimental status in the absence of ZSTK474 but also shift from detrimental state to a restorative state after MCAO in the presence of ZSTK474.

Quantitative real-time PCR produced results consistent with those of double immunofluorescence staining. Compared with the control group, the ZSTK474-treated group expressed a far lower value for the detrimental state’s mRNA markers (CD16, CD32, and CD86), whereas the mRNA markers of restorative state (CD206, YM-1, and Arg 1) were induced dramatically at 72 h (Fig. 3e, f). These data show that ZSTK474 can convert the phenotype of microglia/macrophages to the restorative state, which produces a neuroprotective effect.

### ZSTK474 inhibits pro-inflammation and promotes anti-inflammatory processes

**Fig. 4:**
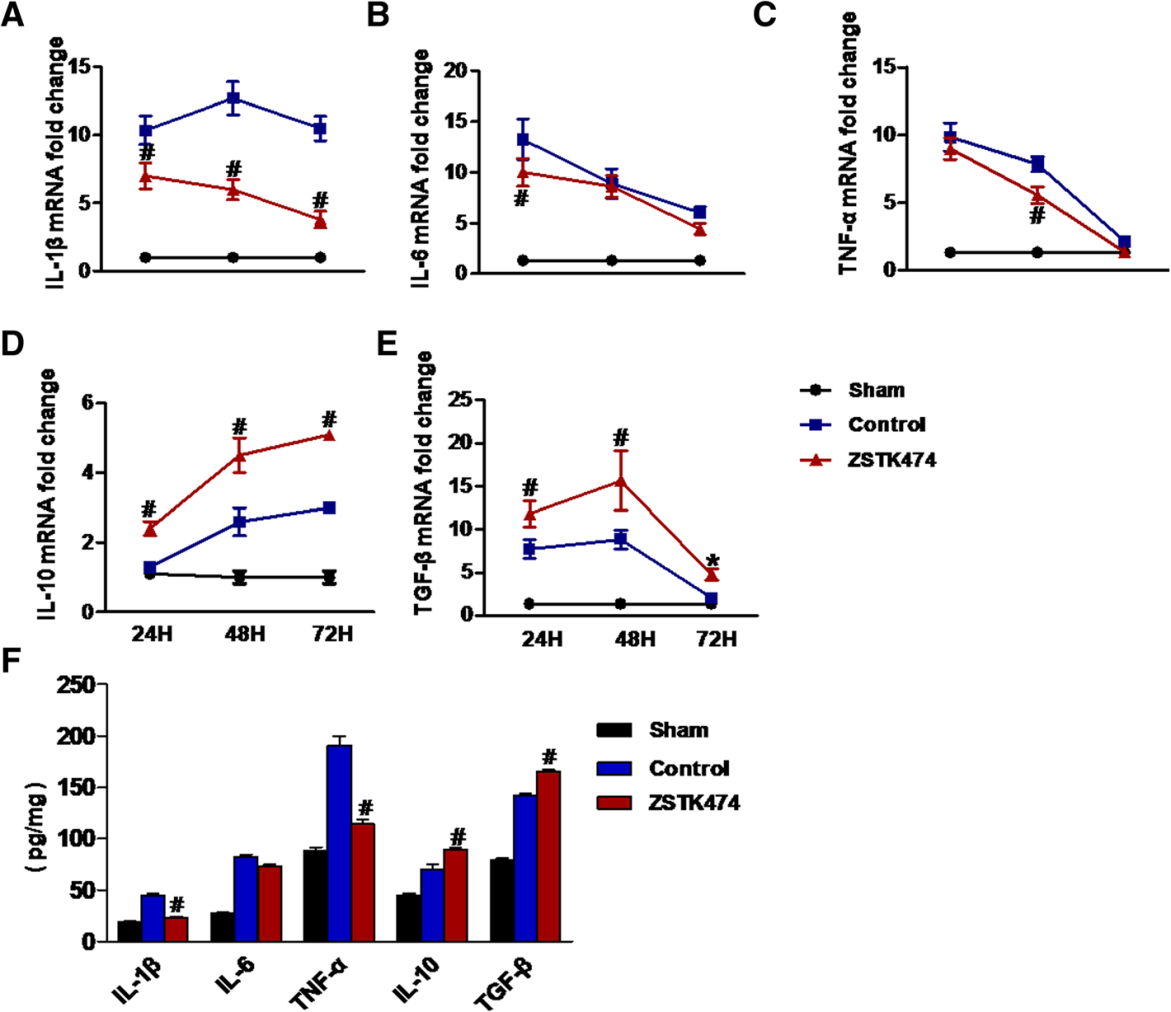
ZSTK474 inhibits pro-inflammatory cytokines and promotes anti-inflammatory cytokines. PCR was performed to quantify the expression of IL-1β (**a**), IL-6 (**b**), TNF-α (**c**), IL-10 (**d**), and TGF-β (**e**) mRNAs in the ischemic region 24, 48, and 72 h of reperfusion. In protein level, the supernatants of brain tissues from mice that underwent 48 h of reperfusion were measured by ELISA kits (**f**). Data in graphs are expressed as means ± SD; ^#^ *P* < 0.001, *P < 0.05 vs. control group; *n* = 6

Secretion of inflammatory cytokines is a key event that follows ischemic stroke. We detected the expression of mRNAs for various inflammatory cytokines (IL-1β, TNF-α, IL-6, IL-10, and TGF-β) in the cortex ipsilateral to sites of ischemia 24, 48, and 72 h after reperfusion (Fig. 4a–e). Generally, expression of these cytokines increased soon after reperfusion. TNF-α and IL-6 expression peaked at 24 h. IL-1β and TGF-β expression peaked at 48 h. IL-10 was rising continuously. However, in the presence of ZSTK474, increases in expression of TNF-α, IL-6, and IL-1β were blunted (Fig. 4a–c). IL-1β expression was most significantly affected by ZSTK474 at 48 h after ischemia reperfusion injury. TNF-α, IL-6, and IL-1β are pro-inflammatory factors that can aggravate the reperfusion injury. TGF-β and IL-10 are anti-inflammatory factors that are regarded as beneficial cytokines, since they reduce inflammation and immune reactions [24]. In our control group, IL-10 expression increased by 2.6-fold at 48 h compared to the sham-operated group (Fig. 4d). ZSTK474 treatment increased IL-10 expression by 4.5-fold compared to that of the control group (Fig. 4d). We also observed higher levels of TGF-β expression in the ZSTK474-treated group at 48 h compared to the control group (Fig. 4e).

According to the results of PCR, we found that control group and ZSTK474-treated group have significant difference at 48 h after MCAO. So, we used ELISA kits to analysis the protein levels of cytokine in the ischemic brain tissues at 48 h of reperfusion. As shown in Fig. 4f, the production of pro-inflammatory cytokines (IL-1β and TNF-α) in the ZSTK474-treated group was significantly reduced compared to the control group. However, there were no marked differences in the levels of IL-6 in the two groups (Fig. 4f). The production of anti-inflammatory cytokines (IL-10 and TGF-β) in the ZSTK474-treated group notably exceeded that in the control group (Fig. 4f).

### ZSTK474-related neuroprotection is mediated by the PI3K/AKT/mTORC1 pathway

**Fig. 5:**
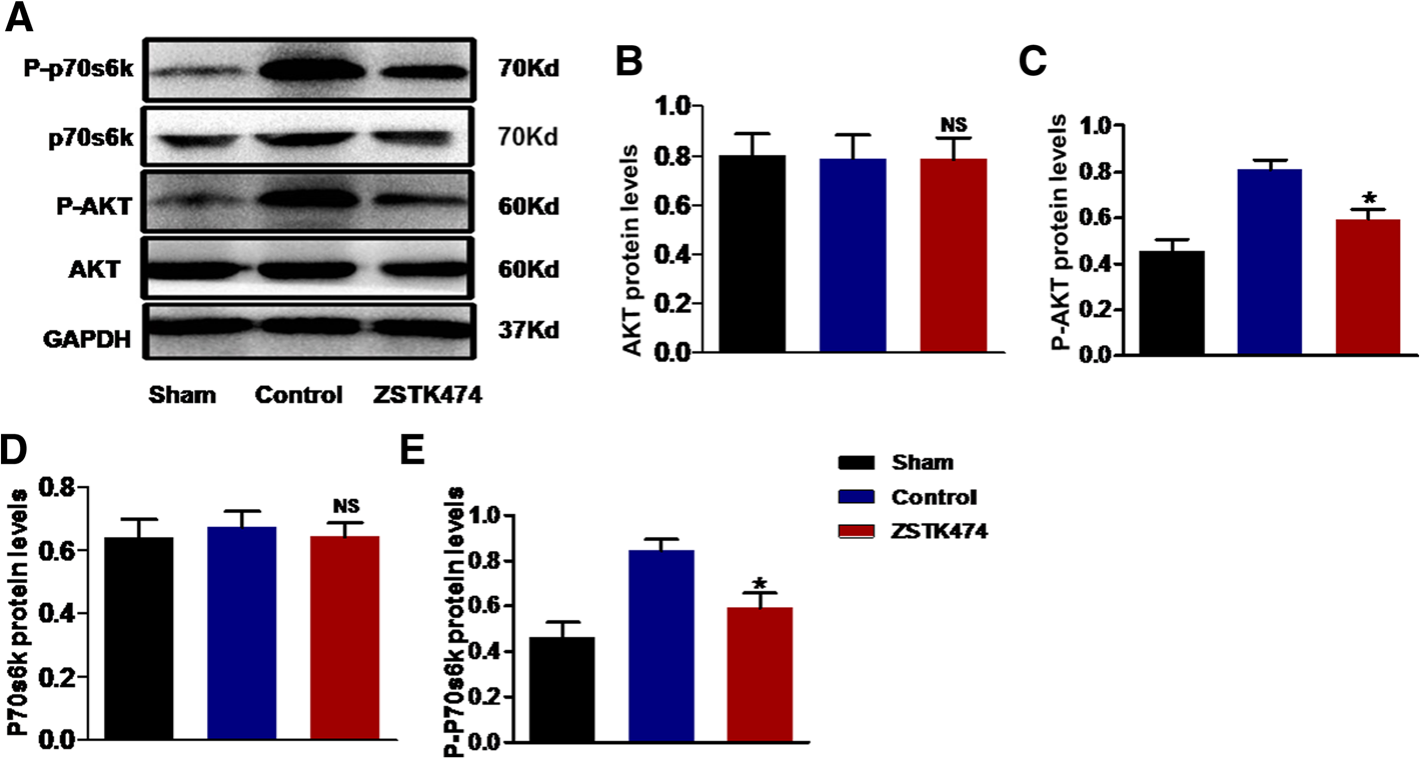
ZSTK474 reduces P-AKT and P-p70S6k protein levels. ZSTK474 suppresses phosphorylation of AKT and other downstream compounds. Western blots (**a**) and densitometry histograms (**b**–**e**) showing AKT, P-AKT, p70S6k, and P-p70S6k protein levels in ZSTK474-treated, control, and sham-treated mice following MCAO. P-AKT and P-p70S6k protein levels were dramatically decreased in the ZSTK474-treated group. Total AKT and p70S6k protein level, however, remained unchanged. Data in graphs are expressed as means ± SD; ^#^ *P* < 0.001, *P < 0.05 vs. control group; NS, not significant; *n* = 6

ZSTK474 inhibits the phosphorylation of AKT, a major target of PI3K, thereby downregulating downstream signaling components. In our study, ZSTK474 treatment decreased the level of phosphorylated AKT (Ser-473) but did not alter total AKT protein levels (Fig. 5a–c). We next assessed whether ZSTK474 treatment affects the levels of mTORC1, a downstream component of the PI3K/AKT/mTORC1 pathway. The mechanism of this effect was ZSTK474’s inhibition of phosphorylation of the p70 ribosomal protein, S6 kinase (p70S6k), on Thr-389 (*P* < 0.05) (Fig. 5a, d, e), confirming that ZSTK474 reduced the level of AKT and p70S6k phosphorylation in this MCAO model.

## Discussion

Microglia/macrophages play a critical role in the inflammatory and immune processes of cerebral ischemia/reperfusion injury; thus, a compound that moderates the activities of microglia and macrophages may have therapeutic potential for preventing otherwise crippling injury [25]. In the present study, we found evidence for the utility of this treatment option by assessing the effect of the PI3K inhibitor, ZSTK474. In mice that experienced ischemic/reperfusion injury, we found that treatment with ZSTK474 inhibited the expression of Iba-1 and GFAP in the ischemic penumbra, altered the phenotype of microglia/macrophages to a restorative state, reduced the production of pro-inflammatory cytokines, increased the production of anti-inflammatory cytokines, and ultimately improved neurological outcome. This original study suggests that ZSTK474 may have neuroprotective power that mitigates cerebral ischemic/reperfusion injury by fostering a beneficial microglial/macrophage phenotype.

Our research indicated that the IPI3K inhibitor, ZSTK474, could mediate the function of microglia/macrophages after ischemia/reperfusion injury. Here, as expected, ZSTK474 treatment diminished the secretion of pro-inflammatory cytokines and enhanced the secretion of anti-inflammatory cytokines at multiple time points. Pro-inflammatory mediators (such as IL-6, IL-1β, and TNF-α) contribute to expanding the area of brain injury in the context of stroke [26, 27]. Indeed, other studies showed that intraventricular injection of TNF-α and IL-1 increased infarct volume and brain edema after MCAO in rats, whereas injection of the microglia inhibitor, minocycline, suppressed microglial activation and the expression of pro-inflammatory cytokines [28]. To the contrary, IL-10 and TGF-β are pleiotropic immunoregulatory cytokines that play a key role in the inflammatory response associated with tissue recovery [24, 29]. Production of the latter cytokines is promoted by phagocytosis and occurs in concert with the removal of dead cells [30]. Here, we found that ZSTK474 treatment not only reduced pro-inflammation production but also increased the levels of TGF-β and IL-10 mRNAs within 24–72 h after ischemic/reperfusion injury.

**Fig. 6:**
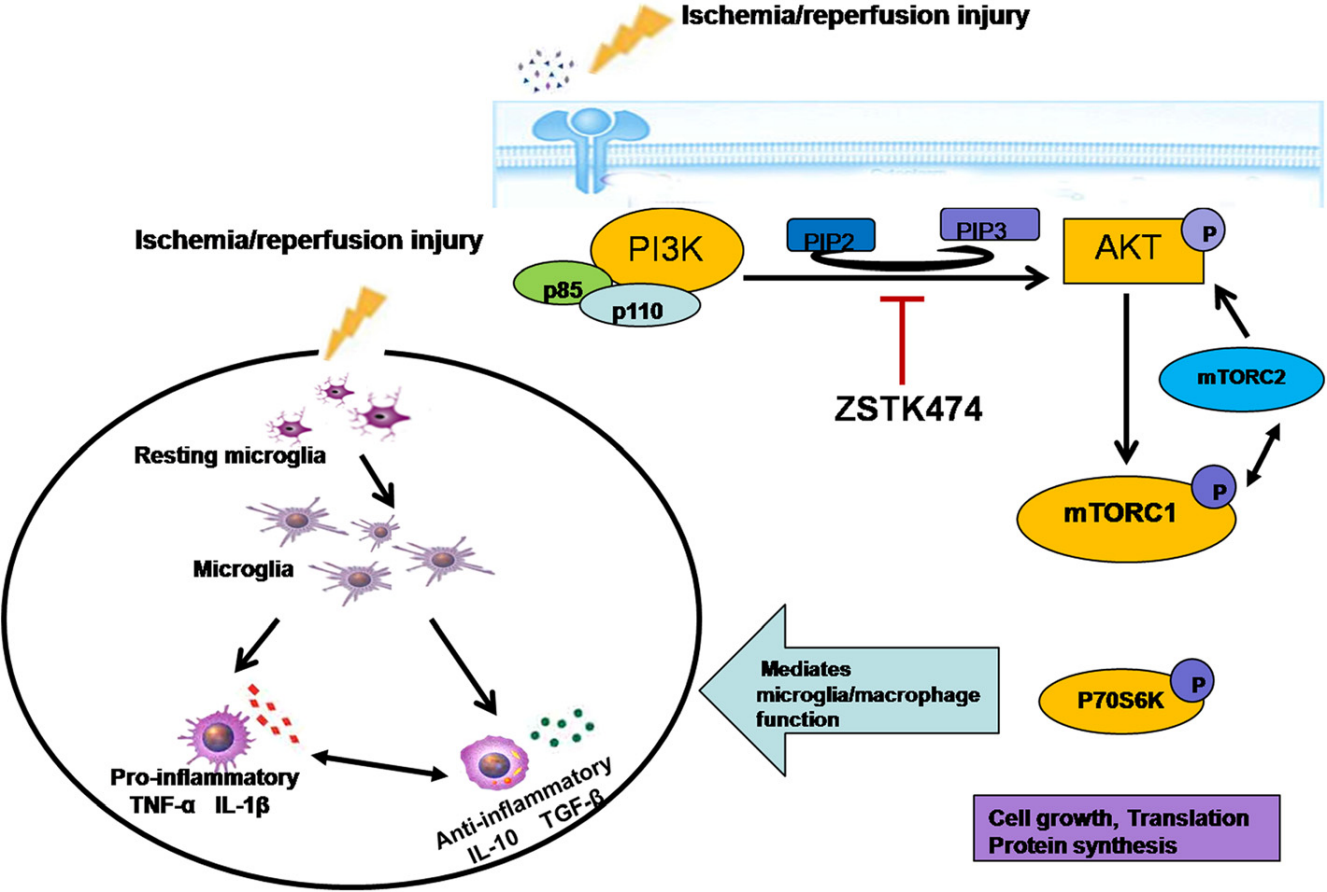
Summary diagram illustrates a hypothesis for how ZSTK474 exerts its effects on ischemic reperfusion injury. Administration of ZSTK474 after cerebral ischemic reperfusion injury decreases the number of microglia/macrophages and shifts their phenotype toward the more restorative state. This treatment reduces the level of pro-inflammatory cytokines and enhances the level of anti-inflammatory cytokines secreted. ZSTK474, a member of a specific class of IPI3K inhibitors, inhibits the phosphorylation of AKT to downregulate downstream mediators. The beneficial effects of ZSTK474 that we observed may result from inhibition of the mTORC1 pathway, which is an important downstream mediator of AKT-adjusted cell growth and translation

AKT is known to control cell growth through the phosphorylation of downstream mTOR complex 1 (mTORC1) which mediates the direct phosphorylation of p70S6k and the leukocyte initiation factor 4E-binding protein [31]. The PI3K/AKT/mTORC1 pathway plays an important role in translation initiation, transcription, cytoskeleton organization, cell growth, proliferation, and cell survival. In accord, our results showed that ZSTK474 inhibited the phosphorylation of AKT and simultaneously reduced phosphorylation levels of p70S6k. This indicates that ZSTK474 may modulate the function of microglia/macrophages through the PI3K/AKT/mTORC1 pathway (Fig. 6). Although some studies performed in vivo and in vitro have shown that the PI3K/AKT/mTORC1 pathway closely relates to microglia/macrophages, the conclusion is controversial [32–34]. The inhibition of mTORC1 is also believed to prevent the microglial/macrophage phenotype toward to a detrimental state, significantly enhancing beneficial anti-inflammatory activity and decreasing the production of pro-inflammatory cytokines and chemokines [33]. Others have suggested that PI3K/AKT, GSK-3, and mTORC1 pathways are potential targets for the development of novel strategies to modulate neuroinflammation [34]. Another point of view is that the upregulation of PI3K/AKT and its downstream pathway could inhibit the activation of microglia in vitro [35, 36]. TP and DAHP are also thought to decrease cell apoptosis in the focal cerebral ischemia of rat brains by a mechanism related to activation of the PI3K/AKT/mTORC1 pathway [37]. Since the cell signal transduction pathway of PI3K/AKT/mTORC1 is a complex and dynamic biological process involving negative feedback, the controversial nature of these conjectures is understandable.

Therefore, we sought a balance in the inflammatory response, the effect of which would resolve these issues. ZSTK474 is a weaker inhibitor of mTORC1 than the PI3K isoforms [9] and its induction of apoptosis is also weaker and cell type-dependent [7]; therefore, we intuited its effect may be more modest. In that framework, the study we designed for using ZSTK474 focused only on the inflammatory response. Certainly, further research will illustrate the important role of PI3K/AKT/mTORC1 and its downstream pathway in microglial/macrophage phenotype.

The present study has some limitations that should be considered. Firstly, PI3Kδ and PI3Kγ are key enzymes in leukocyte signaling and are promising agents for interventions that target signaling pathways involved in inflammatory and autoimmune diseases [38, 39]. ZSTK474 as a pan PI3K inhibitor possesses anti-inflammatory potential that may be related to PI3Kδ and PI3Kγ. But our research focused only on the PI3K/AKT/mTORC1 pathway. Secondly, this study did not fully assess the effects of ZSTK474 on astrocytes, despite evidence indicating that inhibition of astrocyte activation enhances neuronal survival and improves outcomes after cerebral ischemia and related reperfusion injury.

## Conclusions

In conclusion, our results demonstrated that ZSTK474 induced a shift in microglial/macrophage phenotype, inhibited inflammatory responses, reduced infarct volume, and improved neurological function in an experimental model of cerebral ischemia/reperfusion injury. These effects appeared be mediated by the PI3K/AKT/mTORC1 pathway. Our findings established a sound foundation for developing a therapeutic strategy that may benefit the patients who endure ischemic stroke.

## Additional file

Additional file 1: Figure S1. ZSTK474 alleviates neurological deficits in the model of ischemic reperfusion. (DOCX 56 kb)

## Abbreviations

ANOVA: Analysis of variance
CD: Cluster of differentiation
CNS: Central nervous system
ELISA: Enzyme-linked immunosorbent assay
GFAP: Glial fibrillary acidic protein
H&E: Hematoxylin and eosin
i.p.: intraperitoneal
Iba-1: Ionized calcium binding adaptor molecule 1
IL: Interleukin
MCAO: Middle cerebral artery occlusion
mNSS: modified neurological severity score
mTOR: mammalian target of rapamycin
mTORC1: mTOR complex 1
PBS: Phosphate-buffered saline
PCR: Polymerase chain reaction
PI3K: Phosphoinositide 3-kinase
rCBF: regional cerebral blood flow
RIPA: Ristocetin-induced platelet aggregation
SYBR: Snergy bands
TGF: Transforming growth factor
TNF: tumor necrosis factor
TPA: Tissue plasminogen activator
TTC: 2,3,5-tripenyltetrazolium chloride
