# Supplementary material for: Class I PI3K inhibitor ZSTK474 mediates a shift in microglial/macrophage phenotype and inhibits inflammatory response in mice with cerebral ischemia/reperfusion injury
Source: J Neuroinflammation. 2016 Aug 22;13(1):192. doi: 10.1186/s12974-016-0660-1 (PMC4994222; doi:10.1186/s12974-016-0660-1)
Supplement: Additional file 1: Figure S1. — ZSTK474 alleviates neurological deficits in the model of ischemic reperfusion. (DOCX 56 kb) [file 12974_2016_660_MOESM1_ESM.docx]

**Supplemental material**

**Supplemental Figure Legends**

**Supplemental Figure 1. ZSTK474 alleviates neurological deficits in the model of ischemic reperfusion.**

Neurological function was examined in mice after MCAO followed by 24, 48 and 72 h of reperfusion. Mice were randomly assigned to receive different doses of ZSTK474 (50, 100, 200 and 300 mg/kg) to determine the optimum dose. In the groups treated with ZSTK474, neurological function scores were significantly better than control group except the corner-test score, as shown in Figure 1A-D. It showed that neurological function deficiency had alleviated with the increasing dose of ZSTK474. But the animals treated with 300 mg/kg dose showed higher mortality rate and severe adverse effects like diarrhea, weight loss. Those were not observed with 50 mg/kg, 100 mg/kg and 200 mg/kg daily. It seemed that 200 mg/kg can exert more significant effect on ameliorating the symptoms and do not show any adverse effects. Therefore, we applied the dosage of 200 mg/kg, daily in our work. Data expressed as means ± SD; # P<0.01, * P< 0.05, vs. control group; n=6-10 per group.

**Supplemental Figure 1.**

**
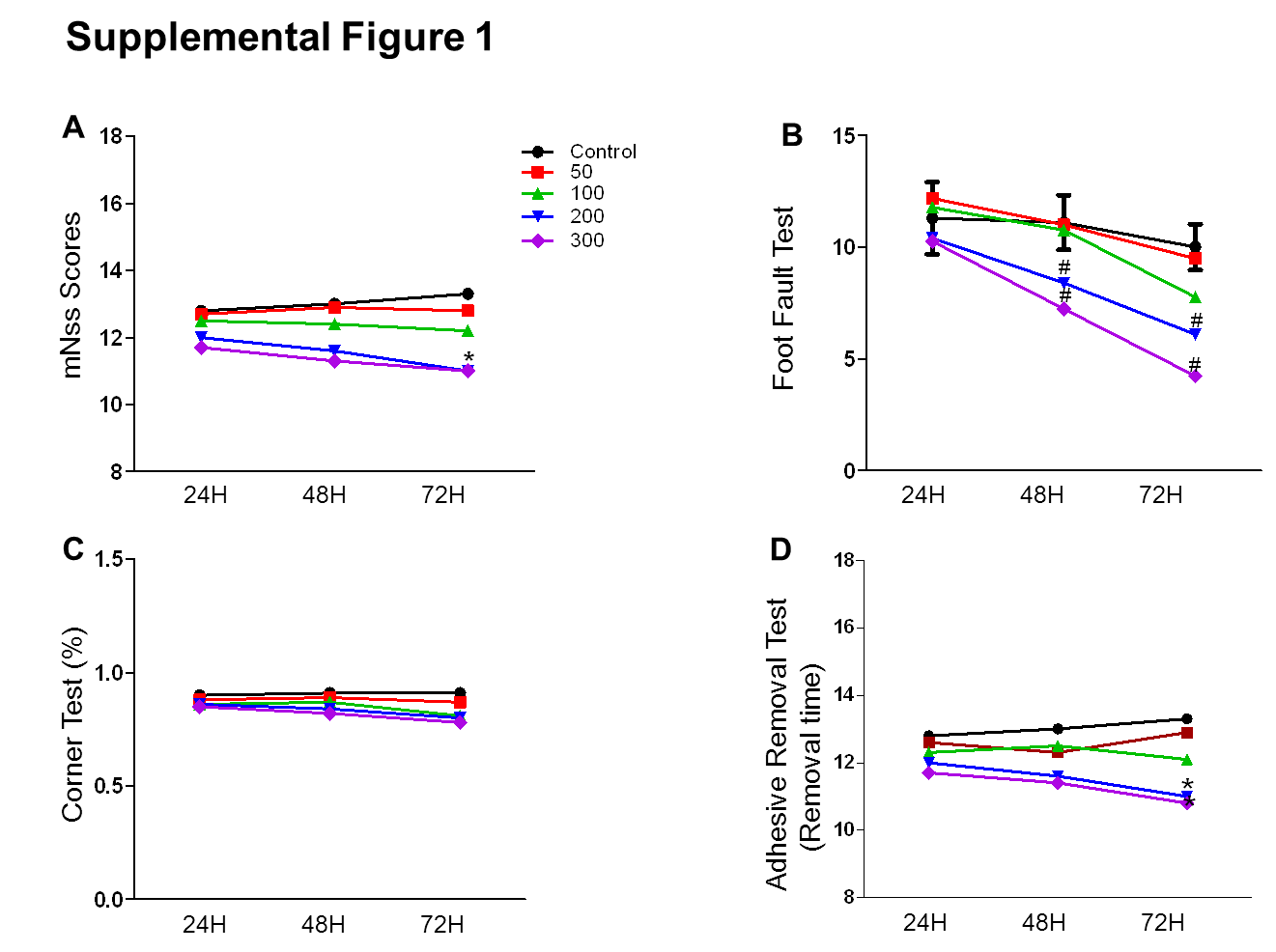
**
